# Evaluation of soluble human leukocyte antigen-G in peripheral blood of pregnant women with gestational diabetes mellitus

**Published:** 2016

**Authors:** Saeide-sadat Shobeiri, Saeid Abediankenari, Bahareh Lashtoo-Aghaee, Zahra Rahmani, Bahareh Esmaeili-gorji

**Affiliations:** 1 Student Research Committee, Mazandaran University of Medical Sciences, sari, Iran**.**; 2 Immunogenetic Research Center, Mazandaran University of Medical Sciences, Sari, Iran**.**; 3 Diabetes Research Center, Mazandaran University of Medical Sciences, Sari, Iran.; 4 Department of Microbiology, School of Medicine, Tehran University of Medical Sciences, Tehran, Iran.; 5 Faculty of Medicine, Mazandaran University of Medical Sciences, Sari, Iran**.**; 6 Department of Biology, Qaemshahr Branch, Islamic Azad University, Qaemshahr, Iran.

**Keywords:** HLA-G, gestational diabetes, pregnancy

## Abstract

**Background:**

Research says that diabetes may develop in over 10% of non-diabetic pregnant women. Diabetes which generally occurs late in second trimester and third trimester of pregnancy, it is called gestational diabetes. Overweight or suffering from obesity before pregnancy is type 2 diabetes risk factor. In most cases, diabetic symptoms disappear after delivery. HLA-G has an important role both in mother and fetus tolerance during pregnancy, it may also be effective in the protection of pancreatic islet cells. In this study, concentration of these molecules in pregnant women with gestational diabetes in comparison with normal pregnant women was investigated.

**Methods:**

In this case-control study, we measured serum HLA-G levels in 24 pregnant women with gestational diabetes compared with 30 normal pregnant women using sandwich ELISA.

**Results:**

HLA-G levels were significantly low in pregnant women with gestational diabetes in contrast to normal pregnant women (P=0.001).

**Conclusion:**

In this study, we found that HLA-G levels were reduced in women with gestational diabetes compared with control group. Therefore, it is suggested that measurement of HLA-G in pregnant women can be considered as an indicator in prognosis of gestational diabetes.

Hyperglycemia of a woman during pregnancy is a special type of diabetes that is called gestational diabetes mellitus (GDM), which is defined as different severities of carbohydrate intolerance first started or diagnosed during pregnancy and is considered as the most common medical complication during pregnancy. Patients can be divided into two categories: those whose diabetes is diagnosed before pregnancy (diabetes before pregnancy or diabetes mellitus) and those whose disease is diagnosed during pregnancy (gestational diabetes) (1). Blood sugar is usually maintained at normal levels by insulin made in the body. In most cases in pregnant women, more insulin is produced to reduce blood sugar levels. Although a number of pregnant women have not been able to do this and suffer from gestational diabetes which mostly occurs in the latter half of pregnancy (2). Women with a history of gestational diabetes, family history of diabetes, premature birth or abortion for an unknown reason, gestational age over 25, high blood pressure, overweight, excessive amounts of amniotic fluid and an infant over 4.5 kg are more susceptible to gestational diabetes (3). Usually within 24 to 28 weeks of pregnancy, glucose tolerance test for diabetes screening is done for pregnant women, but women who have risk factors for this, are examined earlier (4).

Gestational diabetes and impaired glucose tolerance test during pregnancy will lead to an increased risk of prenatal death and progression to diabetes mellitus in 30-50% of women in the future (5).

Gestational diabetes is harmful to both mother and fetus, although there is no evidence of an increased risk of prenatal death in cases that are treated but some studies have shown that the risk of prenatal death increases in cases of untreated gestational diabetes (6).

 Women who are diagnosed with gestational diabetes should regularly monitor their blood sugar levels. Treatment involves keeping maternal blood glucose levels within the normal range and assuring fetal health, careful monitoring of mother and fetus should be done, and a proper diet to keep blood sugar at normal levels must be met. If the diet is not effective, oral diabetes medications or insulin therapy should be done (7). 

All symptoms of diabetes usually disappear after delivery. The cause of gestational diabetes is still unknown, although it is thought that the hormones present in pregnancy, particularly human placental lactogen are associated with gestational diabetes, but the reason why gestational diabetes, occurs in some pregnant women is unknown (8). Human leukocyte antigen-G (HLA-G) is a HLA class Ib molecule that is located on chromosome 6 at l6p21.3 location (9, 10), and plays a major role in tolerance between mother and fetus (11). This molecule was first detected on placental cytotrophoblasts (12). 

In normal conditions, HLA-G molecule is expressed in the thymus, cornea and nail matrix (13-15) but in other tissues, they are expressed in pathological conditions such as tumor lesions, inflammation, autoimmune diseases, and viral infections (16-18). It is possible that the events leading to gestational diabetes have been caused by fetal antigen load. It appears that fetal HLA-G expression which protects the fetus through the down-regulation of cytotoxic T cell to fetal trophoblast, can also be involved in the protection of pancreatic islet cells (8). Therefore, in this study, the role of soluble HLA-G in women with gestational diabetes compared with healthy controls was studied.

## Methods

In this case-control study, the relation between soluble HLA-G with gestational diabetes in pregnant women with gestational diabetes compared with control group was investigated. 24 pregnant women with gestational diabetes confirmed by gynecologist and 30 normal women with the same age and gestational age participated. Inclusion criteria for pregnant woman included: suffering from gestational diabetes in current pregnancy to participate in the study and a history of diabetes before pregnancy and drug use was the exclusion a criteria to leave this study. These items were evaluated using patients` history, checkups and periodic examinations and collecting information through questionnaires with gynecologist and obstetrician approval.

**ELISA (ENZYME-LINKED IMMUNOSORBENT ASSAY):** Two milliliter of blood was collected and after centrifugation serum was extracted. We measured serum soluble HLA-G by commercial ELISA kit (HANGZHOU EASTBIOPHARM, China) according to kit protocol. This kit uses double-antibody sandwich technique to measure serum concentrations of soluble HLA-G in case and control group. Briefly, we prepared five standard solutions with 2400ng / L, 1200ng / L, 600ng / L, 300ng / L, 150ng / L, 75ng / L concentrations by diluting stock 2400ng /L concentration. 

The first well was filled by blank and none of the samples and biotinylated HLA-G antibody were added. Then, we added 50 microliters of standard and 50 micro liters of streptavidin HRP in wells for standards, and in wells related to samples, 40 micro liters of samples plus 10 micro liters of biotinylated HLA-G antibody and 50 micro liters streptavidin-HRP were added to sample wells. Next, the plate was shaken and incubated for 60 min at 37°C. Distilled water was used to dilute 30X washing solution 30 times. Then we washed the plate five times. After washing, 50 microliters of chromogen A and 50 micro liters of chromogen B were added to each well, the plate was a little shaken and was incubated at 37°C for 10 min in the dark and then 50 microliters stop solution was added to each well. Finally, blank was considered zero and optical density (OD) was measured by ELISA reader under 450 nm wavelength. Concentrations of the samples were obtained according to standard concentration and a standard curve that was plotted by the device. We used SPSS Version 19 with Mann-Whitney test to analyze the data.

## Results

Totally, 54 women participated in this study in which 24 of these women are suffering from gestational diabetes with no history of diabetes and also without taking any medications (type A1) as the case group, and 30 normal pregnant women the control group. Gestational age and age of women in control group were matched with case group. The age range of the patients in case group was 24 to 39 years with a mean and standard deviation of 29.8±4.10 and in the women in control group was 23 to 38 years with a mean and standard deviation of 28.9±3.62. Gestational age of pregnant women with GDM was between 27 and 34 weeks and 2 of these pregnant women were under 20 weeks of pregnancy. Demographic characteristics of the study population are shown in table 1.

**Table 1:** Demographic characteristics of pregnant women with gestational diabetes (cases) and normal pregnant women (control)

**Significant level**	**Case** **N=24**	**Control** **N=30**	**Groups** **Variable**
0.401	29.8±4.10	28.9±3.62	Age
0.138	73.93±10.01	74.13±8.62	Weight
0.041	24.32±3.75	21.26±4	Body mass index (BMI)

Statistical analysis showed no significant differences in age and weight between the case group and control group.

**Figure 1:**
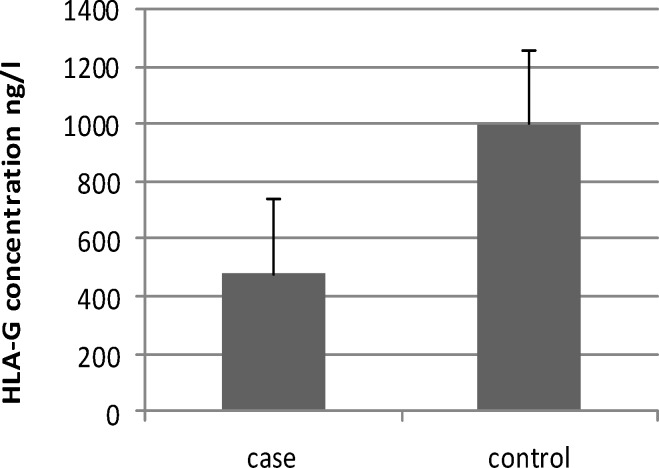
Comparison of HLA-G levels in pregnant women with gestational diabetes and normal pregnant women (479.79±787.26 vs 999.98±340.64

Soluble HLA-G levels decreased in pregnant women with gestational diabetes compared with control group. As shown in figure 1, this difference is considered significant (P=0.001).

## Discussion

In this study, the concentration of soluble HLA-G in pregnant women with gestational diabetes compared with normal pregnant women was studied and a significant decrease in the concentration of these molecules in pregnant women with gestational diabetes compared with normal pregnant group was observed (P=0.001). Many studies have examined the HLA-G soluble in a variety of disorders such as preeclampsia (19), tumors (20), autoimmune diseases (21), and abortion (22); studies have also been conducted in the area of diabetes which mostly included diabetes type I and type II (23, 24). 

In different studies in regard to gestational diabetes and the role of HLA-G *immune tolerance* molecule, recommendations have been made (6, 8), but so far, a study which directly deals with this molecule in gestational diabetes has not been carried out. Carosella et al. in a study assessed the association between HLA-G molecule and autoimmune diseases and showed the role of these molecules in silencing of inflammation in some patients and introduced this molecule as a regulatory factor on the cell surface (21). Hunt et al. examined the soluble HLA-G in the blood of pregnant women compared to nonpregnant women using ELISA method and found that this molecule is present in all stages of pregnancy and its level was significantly higher in pregnant women than non-pregnant women and its value rises with advancing pregnancy (25). 

Vautert et al. examined the intracellular interactions and showed that HLA-G molecules in addition to maternal-fetus tolerance, also play a role in regulating the immune response (26). In the present study, showed that the presence of HLA-G maybe involved in immune regulation, because normal pregnant women due to higher levels of these molecules in their serum compared to pregnant women with diabetes did not suffer from diabetes during pregnancy, in other words, the immunomodulatory of these pregnant women is better and more efficient than women with diabetes. Oztekin O studied the role of HLA-G in gestational diabetes and its development of diabetes type 2, and suggests that HLA-G may even play a role in developing diabetes during pregnancy and even can lead to diabetes type 2 (8). 

In this study, we have shown that HLA-G in normal pregnant women is higher than pregnant women with diabetes, this could be related to the activity of these molecules in regulating the immune system. Higher concentrations of these molecules in normal pregnant women could be indicative of inhibition of the immune response and the regulatory role of this molecule in pregnancy and lower values in pregnant women with gestational diabetes may be an indicative of removal of this inhibition and regulation. It is suggested that this assumption can be the reason for developing gestational diabetes in these women comparing to normal control group. The results of this study showed significant reduction of HLA-G levels in pregnant women with gestational diabetes compared with normal pregnant women. So we can conclude that HLA-G molecule is among the factors for regulation and control of the immune response and the induction of tolerance and can be an important indicator in determining gestational diabetes. Therefore, it is recommended that HLA-G in pregnant women is tested as an effective molecule so as to take an important step in the prediction of gestational diabetes to prevent problems and consequences of this disease.

## Notes

**Funding:** This study was supported by Immunogenetics Research Center, (no. 92-195) Mazandaran University of Medical Sciences.

## Citation:

Shobeiri SS, Abediankenari S, Lashtoo-aghaee B, et al. Evaluation of soluble human leukocyte antigen-G in peripheral blood of pregnant women with gestational diabetes mellitus. Caspian J Intern Med 2016; 7(3): 178-182.

## References

[1] Cunningham FG, Leveno KJ, Bloom SL (2010). Williams obstetrics.

[2] Moyer VA, U.S. Preventive Services Task Force (2014). Screening for gestational diabetes mellitus: US Preventive Services Task Force recommendation statement. Ann Intern Med.

[3] Serlin DC, Lash RW (2009). Diagnosis and management of gestational diabetes mellitus. Am Fam Physician.

[4] American Diabetes Association, Bantle JP, Wylie-Rosett J (2008). Nutrition recommendations and interventions for diabetes: a position statement of the American Diabetes Association. Diabetes Care.

[5] Vambergue A, Fajardy I, Bianchi F (1997). Gestational diabetes mellitus and HLA class II (-DQ, -DR) association: The Digest Study. Eur J Immunogenet.

[6] Kaaja R, Rönnemaa T (2008). Gestational diabetes: pathogenesis and consequences to mother and offspring. Rev Diabet Stud.

[7] Pridjian G, Benjamin TD (2010). Update on gestational diabetes. Obstet Gynecol Clin North Am.

[8] Öztekin Ö (2007). New insights into the pathophysiology of gestational diabetes mellitus: possible role of human leukocyte antigen-G. Med Hypotheses.

[9] Abediankenari S1, Ghasemi M, Kim YJ (2011). Human leukocyte antigen-G expression on dendritic cells induced by transforming growth factor-beta1 and CD4+ T cells proliferation. Iran Biomed J.

[10] Van Der Ven K, Pfeiffer K, Skrablin S (2000). HLA-G polymorphisms and molecule function--questions and more questions--a review. Placenta.

[11] Abediankenari S, Ghasemi M (2009). Generation of immune inhibitory dendritic cells and CD4+T regulatory cells inducing by TGF-beta. Iran J Allergy Asthma Immunol.

[12] Carosella ED, Moreau P, LeMaoult J, Rouas-Freiss N (2008). HLA-G: from biology to clinical benefits. Trends Immunol.

[13] Shakhawat A, Shaikly V, Elzatma E (2010). Interaction between HLA-G and monocyte/ macrophages in human pregnancy. J Reprod Immunol.

[14] Solier C, Aguerre‐Girr M, Lenfant F (2002). Secretion of pro‐apoptotic intron 4‐retaining soluble HLA‐G1 by human villous trophoblast. Eur J Immunol.

[15] Veit TD, Vianna P, Chies JAB (2010). HLA-G-From fetal tolerance to a regulatory molecule in inflammatory diseases. Curr Immunol Rev.

[16] Ito T, Ito N, Saathoff M (2005). Immunology of the human nail apparatus: the nail matrix is a site of relative immune privilege. J Invest Dermatol.

[17] Le Discorde M, Moreau P, Sabatier P, Legeais JM, Carosella ED (2003). Expression of HLA-G in human cornea, an immune-privileged tissue. Hum Immunol.

[18] Mallet V, Blaschitz A, Crisa L (1999). HLA-G in the human thymus: a subpopulation of medullary epithelial but not CD83(+) dendritic cells expresses HLA-G as a membrane-bound and soluble protein. Int Immunol.

[19] Yie SM, Li LH, Li YM, Librach C (2004). HLA-G protein concentrations in maternal serum and placental tissue are decreased in preeclampsia. Am J Obstet Gynecol.

[20] Campoli M, Ferrone S (2008). Tumor escape mechanisms: potential role of soluble HLA antigens and NK cells activating ligands. Tissue Antigens.

[21] Carosella ED, Moreau P, Aractingi S, Rouas-Freiss N (2001). HLA-G: a shield against inflammatory aggression. Trends Immunol.

[22] Farzad F, Abediankenari S, Rahmani Z (2013). The role of HLA-G4 and G5 in threatened-abortion women. J Mazand Univ Med Sci.

[23] Bugawan T, Klitz W, Alejandrino M (2002). The association of specific HLA class I and II alleles with type 1 diabetes among Filipinos. Tissue Antigens.

[24] Solini A, Muscelli E, Stignani M (2010). Soluble human leukocyte antigen-g expression and glucose tolerance in subjects with different degrees of adiposity. J Clin Endocrinol Metab.

[25] Hunt JS, Jadhav L, Chu W, Geraghty DE, Ober C (2000). Soluble HLA-G circulates in maternal blood during pregnancy. Am J Obstet Gynecol.

[26] Hviid TV, Christiansen OB (2005). Linkage disequilibrium between human leukocyte antigen (HLA) class II and HLA-G--possible implications for human reproduction and autoimmune disease. Hum Immunol.

